# Early life exposure to clonazepam has both short- and long-term effects on seizures induced with pentylenetetrazol (PTZ)

**DOI:** 10.3389/fphar.2025.1725780

**Published:** 2026-01-13

**Authors:** Hana Kubova, Grygoriy Tsenov, Anita Conti, Alessandro Negri, Lenka Roubalova, Pavel Mares

**Affiliations:** Institute of Physiology, Academy of Sciences of the Czech Republic, Prague, Czechia

**Keywords:** clonazepam, neonatal rat, pentylenetetrazol seizures, seizure susceptibility, withdrawal phenomena

## Abstract

**Introduction:**

The abrupt cessation of chronic benzodiazepine administration is associated with the development of withdrawal symptoms like increased susceptibility to seizures or seizure development in both animals and humans. Although withdrawal phenomena have been studied in detail in adult animals, information about their development and nature in the immature brain is lacking. Substantial experimental evidence suggests that exposure to BZDs early in life permanently alters brain circuitry and functions. However, the possible long-term modification of seizure propensity has not yet been studied.

**Methods:**

Clonazepam (CZP) was injected into rat pups daily at a dose of 1 mg/kg for five consecutive days, starting on postnatal day 7 (P7) and continuing until P11. Seizure susceptibility was assessed using a pentylenetetrazol (PTZ)-induced seizure model. PTZ induces three types of seizures in rodents that differ by developmental profile and manifestations: convulsive myoclonic seizures (mS) and generalized tonic-clonic seizures (GTCS), and absence-like rhythmic spike-and-wave EEG activity (RMA). Seizures were induced with a single threshold dose of 50–60 mg/kg on days 2, 4, 7, 10, or 14, or with three additive doses of 20 mg/kg on days 7 and 14, or 3 months after the end of treatment. Convulsions accompanying mS and GTCS were detected behaviorally, and RMA was detected in EEG recordings.

**Results:**

The effects of early-life CZP exposure on susceptibility to PTZ-induced seizures were highly dependent on the interval after treatment cessation and the seizure type. Cessation of CZP after a single PTZ threshold dose resulted in an increase in seizure severity compared to controls that was driven by an increased incidence of GTCS lasting 1 week (up to P18). Early-life CZP exposure led to decreased latency to the first RMA and increased RMA frequency after the first PTZ dose of 20 mg/kg in adult (P90) animals, but it did not change RMA parameters in juvenile rats.

**Conclusion:**

Abruptly ceasing clonazepam administration in infant rats results in the development of withdrawal phenomena, represented by a striking increase in seizure propensity. Interestingly, transient augmentation of GABAergic inhibition during critical periods of synaptogenesis and neural network formation and maturation permanently modifies susceptibility to PTZ-induced epileptiform activity.

## 1 Introduction

Since their introduction into clinical practice, benzodiazepines (BZDs) have been among the most widely used drugs, and their stable efficacy throughout development has been documented in both clinical and preclinical studies (for review, see Farrell, 1986; Kubova, 1999). They are used for a wide range of medical indications in all patient age groups, including neonates (Rennie and Boylan, 2003). Because of their use in pediatric patients, the adverse effects and possible disruptive effects of early life BZD exposure to normal brain development are of great concern.

When carefully prescribed, benzodiazepines can be used safely and effectively for short periods (two to 4 weeks) in most patients. However, long-term use increases the risk of serious adverse effects such as tolerance, dependence, and the development of withdrawal syndrome following treatment cessation. Severe withdrawal syndromes, characterized by various neuropsychiatric symptoms, have been reported in patients and laboratory animals treated with benzodiazepines (BZDs) over extended periods (for rev. Lader, 2011; Lader, 2014; Hirschtritt et al., 2021; Baldwin et al., 2013). These symptoms develop shortly after treatment cessation and gradually subside over time. Factors contributing to the appearance of withdrawal symptoms include abrupt discontinuation, alcohol or drug abuse, and age: elderly people and children are particularly vulnerable (Authier et al., 2009). Although various aspects of withdrawal symptoms have been studied in adult animals, the ontogenetic aspects have largely been overlooked and research into this phenomenon in young animals is scarce. The same is true of the age-related differences in mechanisms responsible for the development of withdrawal manifestations. Studies on benzodiazepine administration in the early stages of postnatal development pay more attention to long-term consequences than to acute changes at cellular, molecular, and systems levels. Many rodent studies have demonstrated that administering these drugs after neuronal differentiation but before complete brain maturation (i.e., during the first 3 weeks of life) results in persistent alterations to brain function and structure (for rev. Tucker, 1985; Frieder et al., 1984; Kellogg, 1988). Despite the potent anticonvulsant effects of BZDs, their possible long-lasting effects on brain excitability have not been studied yet.

BZD effects are specifically mediated by their interaction with the BZD receptor binding site, which modulates the efficacy of the primary inhibitory neurotransmitter, gamma-aminobutyric acid (GABA), on GABA_A_ receptors (for rev. Pallanti et al., 2024). Long-lasting enhancement of GABA-mediated inhibition during chronic BZD administration triggers adaptive processes involving both GABAergic and glutamatergic systems (Allison and Pratt, 2003). The impact of BZD exposure on these two major neurotransmitter systems has been studied in detail in adult animals, with only transient changes reported. In our previous studies, we documented dysregulation in the expression of selected glutamatergic and GABA receptor subunit mRNAs, as well as receptor binding, during withdrawal, and in adult animals exposed to clonazepam early postnatally. Comparing the results of these studies with already published data from adult animals suggests that exposure to BZDs alters these receptors differently in immature and mature brains (Kubova et al., 2018; Kubova et al., 2020).

The occurrence of spontaneous seizures and increased susceptibility to seizures induced by various chemical convulsants or electrical stimulation has been documented in adult rodents following abrupt cessation of treatment (Tsuda et al., 1997; Loscher et al., 1996; Izzo et al., 2001; Steppuhn and Turski, 1993). In the present study, we used pentylenetetrazol (PTZ)-induced seizures to evaluate potential changes in excitability across different age groups of animals exposed to BZD early in life. PTZ has been identified as a GABA_A_ receptor inhibitor. Originally, it was hypothesized that PTZ would interact with the same binding site as benzodiazepines (see Mohler et al., 1978). However, subsequent studies have demonstrated that PTZ binds to the pictoxin-binding site in chloride channels of GABA_A_ receptors (Huang et al., 2001). Other mechanisms have also been mentioned sporadically (for a review, see Monteiro et al., 2024).

In laboratory rodents, PTZ has been demonstrated to induce three distinct types of epileptic seizures: two convulsive (myoclonic (mS) and generalized tonic-clonic (GTCS) seizures) (Velisek et al., 1992) and one nonconvulsive (absence-like rhythmic spike and wave EEG activity, RMA) (Zouhar et al., 1980; Schickerova et al., 1989). The convulsive seizures exhibit a correlation with specific behaviors, while the nonconvulsive seizure type manifests as absence-like rhythmic spike and wave activity in EEG, which is reliably detectable only via electroencephalogram (EEG). The development of individual PTZ-seizure types is highly age-dependent. In contrast to GTCS, which were demonstrated already in P4 rats (Vernadakis and Woodbury, 1969), neither mS nor RMA can be reliably induced during the first two postnatal weeks (Mares and Schickerova, 1980; Velisek et al., 1992). PTZ-induced seizures can help detect both BZD-induced developmental delays in brain maturation and seizure-specific patterns of increased susceptibility.

Based on our previous studies demonstrating its profound anticonvulsant and anxiolytic effects in infantile rats, we selected clonazepam (CZP) as a model BZD. We also demonstrated that exposure to CZP during the early stages of development leads to acute and permanent changes in neurotransmission and behavior, though it does not result in substantial growth retardation or developmental delay. (Kubova et al., 2018; Kubova et al., 2020). The present study aims to map changes in response to PTZ and the duration of the withdrawal period after treatment cessation, as well as chronic changes observed long after the end of CZP administration. The study uses the same CZP administration schedule as previous ones. To examine the effects of early-life CZP exposure on different seizure types, two PTZ administration schemes were employed: a single subcutaneous administration of the threshold PTZ dose in infantile and juvenile animals, and an additive intraperitoneal administration of a subthreshold PTZ dose in juvenile and adult rats.

## 2 Methods

### 2.1 Animals

Experiments were performed in male Wistar albino rats (Institute of Physiology, Academy of Sciences, Prague). In total, 166 pups were used for this study. Two animals were excluded because of technical problems with EEG recording; one has to be euthanized as adult because of tumor and two animals died in connection with the CZP treatment. The day of birth was counted as zero (P0). Rats were housed in a controlled environment (temperature 22 ± 1 ^o^C, humidity 50%–60%, lights on 0600–1800 h) with free access to food and water. At P5 number of pups was reduced and animals were fostered so that each litter consisted of 10 pups. Before CZP administration, pups in each were randomly divided into two groups: controls (n = 5) and CZP treated (n = 5). All procedures involving animals and their care were conducted according the ARRIVE guidelines in compliance with national (Act No 246/1992 Coll.) and international laws and policies (EEC Council Directive 86/609, OJ L 358, 1, 12 December 1987; Guide for the Care and Use of Laboratory Animals, U.S. National Research Council, 1996). The Ethical Committee of the Czech Academy of Sciences approved the experimental protocol (Approval No. 35-2022P). All experiments were performed in a blind manner, with the experimenter unaware of the treatment received by each animal. The experimental groups consisted of 8–18 animals. Figures 2–4 (Panel A) show the number of animals in each group. This number was determined based on previous experiments with the PTZ model and calculated using a power calculation for an unpaired t-test with data from previous experiments using the same PTZ models. Sample sizes were found to range between 8 and 19 animals per group.

For extrapolation of experimental data to human studies it is critical to know how the age of animals correlate with that of humans. Various parameters have been used for comparisons. Dobbing (1970) reported that the timing of a “growth spurt” occurring in human babies between the last few weeks of gestation and the first few months of life corresponds with that of rats aged P10-12. In addition to biochemical parameters used by Dobbing, development of electrical activity of the rat brain has shown that irregular EEG activity appears at the age of 5–6 days (Ellingson and Rose, 1970; Mares et al., 1979). EEG activity is up to P10 interrupted by periods of electrical silence corresponding to “tracé alternant” described in preterm newborns but never seen in full-term human newborns (Ellingson, 1964).

### 2.2 Pharmacological treatment

CZP was suspended in physiological saline supplemented with Tween 80 (CZP 1 mg/5 mL of saline and one drop of Tween 80) and injected intraperitoneally at a dose of 1 mg/kg/day for 5 consecutive days beginning on P7 until P11. As reported previously, a single administration of the selected dose of CZP exerted anticonvulsant effects for more than 24 h in immature rats subjected to the pentylenetetrazol (PTZ) model (Kubova and Mares, 1989; Mikulecka et al., 2011). Control animals received solvent instead of CZP. After injection, the pups were immediately returned to their dams. Separation from the mother during drug administration never exceeded 20 min. Throughout drug administration and all experiments, the pups 18 days old and younger were maintained at 34 °C ± 2 °C using an electric heating pad connected to a digital thermometer to compensate for the immature thermoregulatory function at this age (Conklin and Heggeness, 1971).

The body weight was checked daily from P6 until P15 and then before pentylenetetrazol administration. To minimize the effects of variability in individual groups, the data was used to calculate relative body weight. The difference in the relative body weights between two consecutive days was used as a measure of weight gain. Maternal behavior was observed after the drug and/or solvent administration for 5 min by an experienced observer.

### 2.3 Effects of repeated CZP administration on PTZ-induced seizures in juvenile and adult animals

The effects of early life CZP exposure on susceptibility to PTZ-induced seizures were assessed in two experimental paradigms:

#### 2.3.1 mS and GTCS seizures induced with threshold dose of PTZ

The experimental schedule is shown in Figure 2, Panel A. The threshold dose of PTZ used for this experiment (50 mg/kg in P13 to P18 and 60 mg/kg in P21 and P25) was selected according to our previous study on age dependent changes in sensitivity to PTZ (Velisek et al., 1992; Kubova, 2009). PTZ (PTZ, Sigma #P6500) was freshly dissolved in physiological saline (50 and/or 60 mg/mL) and injected subcutaneously on P13, 15, 18, 21 and/or P25. After PTZ injection, the animals were individually placed in a Plexiglas cage, and their behavior was monitored for 30 min by an experienced observer. Until P18, animals were maintained at 34 ± 2 ^o^C with a Physiological-Biological Temperature Controller (TMP-5b; Supertech; Hungary) to compensate for the immature thermoregulation at this age (Conklin and Heggeness, 1971). The incidence and latency of two types of motor convulsions, *myoclonic seizures* (mS; predominantly involving the head and forelimb muscles with preserved righting reflexes) and *generalized tonic-clonic seizures* with or without tonic phase (GTCS or GCS; initiating as a short running phase and accompanied by a loss of righting reflexes), were registered. To assess the severity of the epileptic phenomena, the animals were assigned a score according to the most severe behavioral characteristics as follows (Pohl and Mares, 1987):

0 - no changes
0.5 - abnormal behavior (e.g., automatisms, increased orienting reactions)
1 - isolated myoclonic jerks
2 - atypical *mS,* i.e., only some phenomena present
3 - clonic seizures involving head and forelimb muscles with preserved righting reflexes
(*mS*)
4 - generalized seizures without the tonic phase (GCS)
5 - complete GTCS with a tonic phase longer than 5 s (GTCS).

#### 2.3.2 RMA and mS induced with additive dose of PTZ

At P18, P25 and P90 animals were implanted with epidural cortical electrodes. Under ether anesthesia, the silver recording electrodes were placed bilaterally over the sensorimotor and occipital areas (Paxinos and Watson, 1986) of both hemispheres. Both the reference and ground electrodes were placed in the occipital bone. Electrodes were fixed to the skull using fast-curing dental acrylic. After a recovery period, the animals were placed individually in Plexiglas cages (15 × 15 cm, 30 cm high), and the electrodes were connected to an EEG monitoring system. EEG data were preamplified (#RA4PA, Tucker-Davis Technologies, Alachua, United States) and recorded (#RX5-Pentuza base station, TDT, United States) using a bandpass of 1.2–300 Hz and a digitization rate of 1 kHz. The baseline EEG was recorded continuously for at least 10 min prior to the first PTZ injection. PTZ was administered intraperitoneally in three consecutive doses of 20 mg/kg with an interval of 20 min between the injections or until the onset of myoclonic seizures. The dosing of PTZ and the intervals between injections were based on the results of previous studies. (Tchekalarova et al., 2013; Brima et al., 2013). The latency, incidence, number and duration of episodes of *RMA* defined as at smallest of the three waves of 4–6 Hz frequency (Brima et al., 2013) were evaluated between 10 and 15 min after the first and second injections. The incidence and latency of mS were also registered. Experimental groups consisted of 8–10 animals and number of animals in individual groups are in Experimental schemes (Figure 2, panel A; Figure 3, panel A; Figure 4, panel A).

## 3 Statistics

Sample size was determined in advance based on previous experience and following the principles of the 3 Rs (Replacement, Reduction and Refinement; https://www.nc3rs.org.uk/the-3rs). At the beginning of experiment, individual animals from each litter were randomly allocated to a particular treatment and interval group. All efforts were made to minimize the number of animals used and their suffering. Data analysis was done blindly to the treatment. The treatment and time points for each group consisted of six to fourteen animals. Data were analyzed using GraphPad Prism 9.5.1 (GraphPad Software, United States) software. Using the D’Agostino-Pearson normality test, all data sets were first analyzed to determine whether the values were derived from a Gaussian distribution. The relative body weight gains were analyzed with Mixed effects analysis followed by FDR-corrected multiple comparison tests. Behavioral seizure scores were analyzed using Kruskal-Wallis test corrected for multiple comparisons by controlling the False Discovery Rate of Benjamini, Krieger, and Yekutieli (Q = 0.05). Latencies to the onset of the 1^st^ RMA and/or mS and duration of mS were analyzed using One-way ANOVA followed by FDR-corrected multiple comparison tests. The number and total duration of RMA was compared with Two-way ANOVA RM followed by FDR-corrected multiple comparison tests. The incidence of minimal seizures as well as GTCS was analyzed using Fisher’s exact test (Holm-Sidak corrected). Probability values <0.05 were considered statistically significant.

## 4 Results

**FIGURE 1:**
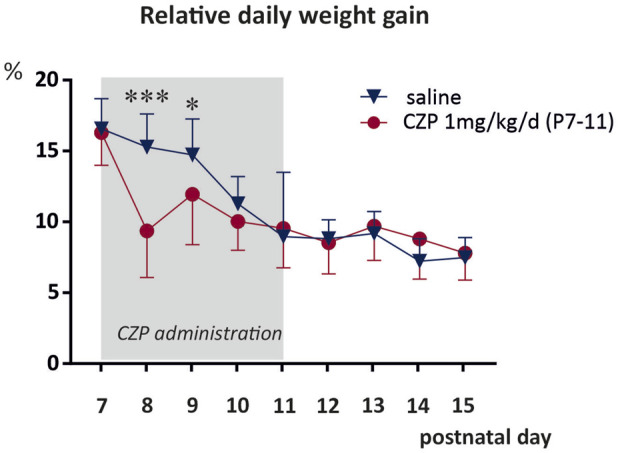
Relative daily weight gain between P7 and P15. Clonazepam (1 mg/kg/day) was administered for five consecutive days, starting at P7. Body weight at P6 was normalised to 100%, and the difference in relative body weight between two consecutive days as a percentage is shown on the y-axis. Relative body weight gains were analysed using mixed-effects analysis, followed by FDR-corrected multiple comparison tests (q < 0.05 taken as discovery). The data show a significant decrease in weight gain compared to the control group during the first 2 days of CZP administration (P7/8, *q < 0.0001*; P8/9, *q = 0.0234*). No differences were observed at later time points. Details of statistical analysis are summarized in Supplementary table A. The x-axis shows age in days. Blue triangles denote solvent-treated controls and red circles denote clonazepam (CZP)-treated rats. Asterisks denote a significant difference from the controls.

Effect of CZP exposure on maternal behavior and body growth (Figure 1, Supplementary Table A).

Regular inspection of the home cages during the experiments revealed that the CZP-exposed pups were not maternally deprived. After CZP administration, the pups were quickly returned to their home cages. The dams showed immediate maternal responsiveness, i.e., they retrieved all pups to the nest site, licked them and assumed a nursing position over them. Administration of CZP significantly decreased the relative body weight gain at P8 and P9 by 38% and 16%, respectively. Starting at P10, weight gain did not differ between controls and CZP exposed animals (Figure 1). No differences in body weight were observed after the CZP cessation at any interval analyzed. Two animals died in connection with the CZP treatment. All statistical details and results of statistical analysis are summarized in Supplementary Table A).

**FIGURE 2:**
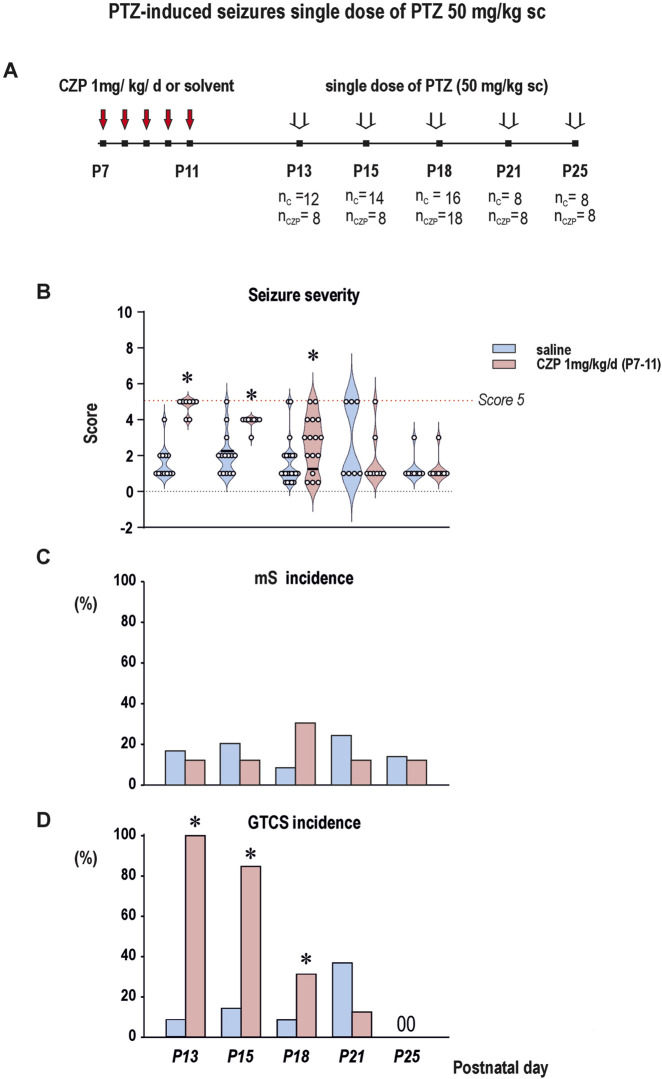
The effects of CZP withdrawal on pentylenetetrazol (PTZ)-elicited seizures in P13, P15, P18, P21 and P25 animals (i.e. 2, 4, 7, 10 and 24 days after the last CZP dose) (x-axis). Clonazepam (1 mg/kg/day) was administered for 5 consecutive days starting at P7. PTZ was administered subcutaneously in a single threshold dose of 50 mg/kg in P13 – P18 and 60 mg/kg in P21 and P25 animals. Experimental groups consisted of 8–18 animals. **(A)** Scheme of the experiment. The figure shows the interval of CZP administration (P7-P11) and the age of the animals at the administration of the threshold dose of PTZ. The number of animals in each age group is specified (n_C_–number of animals in the control group, n_CZP_–number of animals in the CZP group). **(B)** Behavioral seizure scores were analyzed using Kruskal-Wallis test corrected for multiple comparisons by controlling the False Discovery Rate of Benjamini, Krieger, and Yekutieli (discovery q < 0.05) and results of analysis revealed that withdrawal of CZP treatment resulted in significant increase of seizure severity expressed as a score (y-axis) up to 7 days after the end of treatment (i.e.,.at P13, *q* = *0.0006;* P15, *q = 0.0422* and P18, *q = 0.0422* animals). This increase was driven by increase incidence of generalized tonic-clonic seizures (GTCS). Dashed line marks Score 5, the level of maximal seizure severity. Graph is presented as violin plots (the sample median and the first and third quartiles). **(C)** Administration of PTZ in threshold doses (50 or 60 mg/kg sc) rarely elicited myoclonic (minimal) seizures (y-axis in %) both in vehicle treated and CZP-exposed animals and the incidence of mS did not differ among age groups (χ2 test p > 0.05 in both controls and CZP exposed animals). **(D)** After CZP cessation, the incidence of GTCS (y-axis) was significantly increased till 7 days after withdrawal (i.e., up to P18) compared to controls. (χ2 test; p = 0.0025). Circles mark individual values. Asterix denotes significant difference compared to controls. Details of statistical analysis are summarized in Supplementary Tables SB1, SB2.

Immediate increase of seizure susceptibility after CZP cessation - mS and GTCS seizures induced with single, threshold dose of PTZ (Figure 2; Supplementary Tables SB1, SB2)).

Statistical analysis did not reveal any significant age-dependent differences in severity and seizure incidence among vehicle treated animals (Figure 2, panel B). The incidence of GTCS was very low and did not differ among age groups (χ2 test 1.723, df = 3; p > 0.05) (Figure 2, panel D). Early CZP exposure significantly increased the severity of seizures induced after treatment cessation with the threshold dose of PTZ until P18 (i.e., seven days after the last CZP injection) (Figure 2, panel D). After that decreased gradually with interval after the treatment cessation (statistics in Supplementary Table SB1). Increase of seizure severity was driven by an increase in the incidence of GTCS (χ2 test 30.35, df = 12; p = 0.0025) (Figure 2, panel B). Compared to the vehicle-treated controls, the incidence of GTCS in the CZP-exposed animals on P13, P15 and P18 increased by 85.7, 67.5% and 34.8%, respectively (Figure 2, panel D) (all statistical details and results of statistical analysis are summarized in Supplementary Table SB2). Like the seizure severity, the incidence of GTCS decreased with time, and beginning on P21 (i.e., 14 days after the final CZP injection), these two parameters did not differ from the control values. Administration of PTZ in threshold doses (50 or 60 mg/kg sc) rarely elicited mS both in vehicle treated and CZP-exposed animals and the incidence of mS in both groups ranged between 9 and 31% and did not differ among age groups (χ2 test 1.899, df = 4; p > 0.05 in controls and 2.462, df = 4; p > 0.05) (Figure 2, panel C).

**FIGURE 3:**
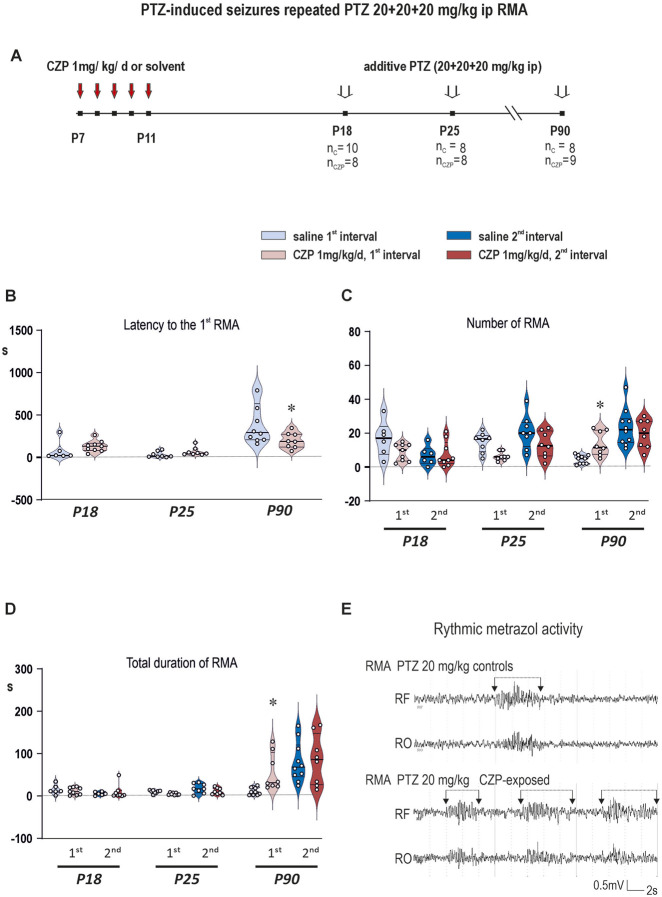
The effects of CZP withdrawal on PTZ-elicited EEG Rhythmic Metrazol Activity (RMA) in P18, P25 and/or P90 rats (i.e. 7 or 14 days or 3 months after treatment cessation) (x-axis, age in postnatal days). Clonazepam (1 mg/kg/day) was administered for 5 consecutive days starting at P7. PTZ was administered intraperitoneally in three consecutive doses of 20 mg/kg with an interval of 20 min between the injections or until the onset of myoclonic seizures to animals with implanted silver recording electrodes placed bilaterally over the sensorimotor and occipital areas. Parameters of RMA epochs were evaluated between 10 and 15 min after the first and second PTZ injections. RMA parameters in the third evaluated interval were not statistically analyzed, because most juvenile animals (75%–100%) have already developed mS after the second dose of PTZ. **(A)** Scheme of the experiment. The figure shows the interval of CZP administration (P7-P11) and the age of the animals at the administration of the threshold dose of PTZ. The number of animals in each age group is specified (n_C_–number of animals in the control group, n_CZP_–number of animals in the CZP group). **(B)** As revealed with One-way ANOVA followed by FDR-corrected multiple comparison tests, latencies to the 1^st^ RMA epoch (y-axis in seconds) were significantly shorter in P90 animals exposed to CZP early in development (*q = 0.0037*). No effect of early life CZP exposure on latencies to the 1^st^ RMA epoch was detected in juvenile rats. **(C)** Number of RMA epochs in evaluated intervals (y-axis) in individual age groups (x-axis, the first row) after the 1^st^ and 2^nd^ administration of PTZ (20 mg/kg each) (x-axis, the second row). Data was analyzed using Two-way repeated measures ANOVA with one between-group factor (treatment) and one within-subject factor (repeated session) followed by FDR-corrected multiple comparison tests (q < 0.05 taken as discovery). In P90 animals after the 1^st^ PTZ administration exposed to CZP early in life number of RMA epochs was significantly higher than in controls (*q = 0.0313*). No effect of early life CZP exposure on this parameter was observed in juvenile animals. **(D)** As revealed with Two-way repeated measures ANOVA with one between-group factor (treatment) and one within-subject factor (repeated session) followed by FDR-corrected multiple comparison tests (q < 0.05 taken as discovery), total duration of RMA activity in seconds (y-axis) after the 1^st^ and 2^nd^ PTZ administration demonstrates significantly prolonged total RMA duration in P90 CZP-exposed animals after the 1^st^ PTZ dose (*q = 0.0030*). Details of statistical analysis are summarized in Supplementary Tables SC1–SC3. Other details as in Figure 3. **(E)** An example of rhythmic metrazol activity (marked with a line segment) after the first PTZ dose in P90 animals. Controls are at the top and CZP-exposed animals at the bottom. RF: the right frontal cortex; RO: the right occipital cortex.

**FIGURE 4:**
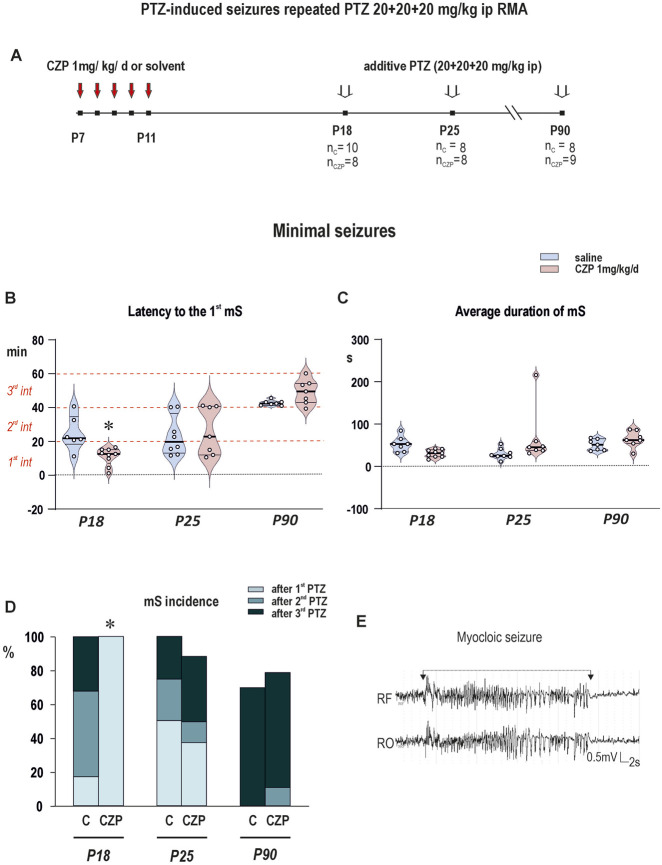
The effect of early life CZP exposure (1 mg/kg daily between P7 and P11) on the development of myoclonic (minimal) seizures induced with repeated intraperitoneal PTZ administration. PTZ was administered intraperitoneally in three consecutive doses of 20 mg/kg with an interval of 20 min between the injections or until the onset of myoclonic seizures. **(A)** Scheme of the experiment. The figure shows the interval of CZP administration (P7-P11) and the age of the animals at the administration of the threshold dose of PTZ. The number of animals in each age group is specified (n_C_–number of animals in the control group, n_CZP_–number of animals in the CZP group). **(B)** Based on results of One-way ANOVA followed by FDR-corrected multiple comparison tests (discovery q < 0.05), graph B illustrates significant shortening of latency to the 1^st^ myoclonic seizure (y-axis, in seconds) in P18 CZP- exposed animals (i.e., one week after treatment cessation) compared to controls (*q = 0.0031*). **(C)** Graph shows that early life CZP exposure had no effect on the duration of myoclonic seizures (y-axis, in seconds) in individual age groups of animals. Details of statistical analysis are summarized in Supplementary Table D **(D)** Graph shows the percentage (y-axis) of animals developing the first myoclonic seizure (mS) after the 1^st^ (pale blue), 2^nd^ (medium blue) and 3^rd^ (dark blue) administration of PTZ (each dose of 20 mg/kg with an interval of 20 min between the injections). Compared to vehicle treated controls, significantly higher percentage of CZP-exposed P18 animals developed mS already after the 1^st^ PTZ (20 mg/kg) (Fisher’s exact test, p = 0.002). Other details as in Figure 3. **(E)** An example of myoclonic (minimal) seizure (marked with line segment) RF–the frontal cortex (right); RO the occipital cortex (right).

Short- and long-term changes in seizure susceptibility - RMA (Figure 3; Supplementary Tables SC1–SC3) and mS (Figure 4; Supplementary Table D) induced with additive dose of PTZ.

Only data from the 1^st^ and 2^nd^ evaluated intervals (i.e., after the 1^st^ and 2^nd^ dose of PTZ) were used for statistical analysis. RMA parameters in the third evaluated interval were not statistically analyzed, because most juvenile animals (75%–100%) have already developed mS after the second dose of PTZ (Figure 4, panel D).

In controls, RMA parameters such as number and total duration of RMA epochs were dependent on the age of the animals on the day of recording and the total dose of PTZ (i.e., the number of the recording session) (Figure 3, panels D and C). As illustrated in Figure 3 in panels D and C, in P90 animals, the number of RMA epochs increased five times between the 1^st^ and 2^nd^ evaluated intervals (4.5 ± 2.6 vs. 23.3 ± 10.9 RMA epochs per monitored interval) and was accompanied by an increase in total duration of RMA activity in evaluated interval (11.6 ± 9.0 vs. 80.2 ± 47.1 s). In contrast, the opposite trend was observed in P18 animals, where the number of RMA episodes was significantly higher in the first than in the second evaluated interval (16.7 ± 10.4 vs. 6.7 ± 5.6 epochs per monitored interval). In the first evaluated interval, RMA episodes were significantly more frequent (16.7 ± 10.4 in P18, 14.5 ± 5.7 in P25 vs. 4.5 ± 2.6 in P90) in both groups of juveniles compared to P90 rats. Latency to the 1^st^ mS was significantly shorter in both juvenile groups compared to adults, which all developed myoclonic seizures (mS) after the 3^rd^ dose of PTZ (1,491 ± 619 in P18, 1,391 ± 695 in P25 vs. 2,561 ± 92 s in P90) (Figure 4, panel B). These data support the results of previous studies demonstrating the higher susceptibility to PTZ in immature animals (Velisek et al., 1992). All statistical details and results of statistical analysis are summarized in Supplementary Tables SC1–SC3 and in Supplementary Table D.

Effects of neonatal exposure to CZP on evaluated RMA parameters were primarily dependent on the treatment and age of animals on the day of recording (i.e., interval after treatment cessation) and their interaction. Interestingly, statistical analysis revealed a significant increase in PTZ susceptibility after the first PTZ dose (20 mg/kg i. p.) in P90 animals. In animals exposed to CZP early in life, the number of RMA epochs was three times higher (13.7 ± 7.2 RMA epochs per the 1^st^ monitored interval compared to 4.5 ± 2.5 RMA epochs in vehicle treated controls) (Figure 3, panel C), the latency to the 1^st^ RMA was 50% shorter (200 ± 92 vs. 400 ± 242 s) and total duration of RMAs was five time longer (59.1 ± 43.6 vs. 11.6 ± 9.0 s) (Figure 3, panel B and D) compared to vehicle treated rats. No differences between CZP and vehicle treated rats occurred in animals monitored at P18 and P25 (Figure 3).

Early life exposure to CZP shortened significantly the latency to the first mS by 58% 1 week after treatment cessation (in P18) (665 ± 310 vs. 1,491 ± 619 s) (Figure 4, panel C) i.e., mS developed after the 1^st^ dose of PTZ in 100% CZP exposed animals (Fisher’s exact test, p = 0.002) (Figure 4, panel D). All statistical details and results of statistical analysis are summarized in Supplementary Tables SC1–SC3 in Supplementary Table D.

## 5 Discussion

An increase in susceptibility to chemically acquired seizures after the cessation benzodiazepine administration has been reported previously in adult rats by others (Rundfeldt et al., 1995; Loscher et al., 1996; Izzo et al., 2001; Mori et al., 2012). Our data demonstrated that withdrawal from CZP results in sensitization to PTZ-induced convulsions for 1 week after the final dosage. In our study, alterations of susceptibility to PTZ seizures depended on interval after the treatment cessation and were related to the PTZ administration schemes and interestingly on seizure type. In contrast to our study, most studies on adult rodents looked at an increase in seizure susceptibility during BZDs withdrawal, without focusing on the different types of seizure. Studies of PTZ sensitivity in adult rodents during the BZD withdrawal period produced conflicting results. Although some authors did not find any change in PTZ sensitivity after abrupt cessation of chronic BZD treatment (Ward and Stephens, 1998), a transient decrease in the PTZ threshold and an increase in the susceptibility to PTZ-induced seizures were reported by many others (Dunworth et al., 2000; Mori et al., 2012; Nidhi et al., 2000; Tsuda et al., 1997; Rundfeldt et al., 1995; Byrnes et al., 1993). The results of these studies are, however, not directly comparable because different schemes of drug administration and dose ranges were used. It must also be emphasized that various BZDs were used, and the differences in their pharmacodynamic and pharmacokinetic properties as well as differences in the experimental designs may substantially alter the functional consequences of chronic BZD administration.

We showed before that giving one dose of 1 mg/kg of CZP to P7 and P12 animals caused a rebound increase in PTZ sensitivity. This was shown by an increase in GTCS cases 48 h later (Kubova and Mares, 1989; Mikulecka et al., 2011). Development of rebound phenomena has not been reported after a single dose of benzodiazepines in adult rodents. In line with pediatric experience (Authier et al., 2009), this may suggest that immature individuals are at higher risk of seizures during BZD-withdrawal period.

In infantile animals exposed to CZP for 5 days, a single administration of the PTZ in threshold dose (50 and/or 60 mg/kg) increased the incidence of generalized tonic-clonic seizures (GTCS) after drug cessation, yet it did not affect the incidence of myoclonic seizures (mS). As previously stated, these two seizure types differ in terms of their ontogeny (Velisek et al., 1992), their sensitivity to anti-seizure medications (Kubova, 1999), and the brain structures involved in their onset (Browning and Nelson, 1986; Faingold, 1987). Despite the absence of direct evidence for molecular mechanisms specific to the development of individual seizure types, neuropharmacological dissociation suggests that mS and GTCS are controlled by distinct molecular and neural substrates. Further study is required to elucidate the mechanisms responsible for the divergent effects of CZP cessation on specific seizure types.

In the second experimental paradigm, involving the administration of additive subthreshold PTZ dosage, it was observed that all P18 animals that had been exposed to CZP earlier in life developed mS after the first PTZ administration (20 mg/kg). The observed discrepancy between the two PTZ models can be explained by the short latencies to GTCS in CZP-exposed animals. In the PTZ model, myoclonic seizures precede GTCS onset (Velisek et al., 1992), and shortened GTCS latency can overlap with myoclonic seizure development. A lower dose of PTZ does not result in the development of GTCS and a decrease in dosage can assist in the unmasking of changes in the effect on susceptibility to myoclonic seizures.

The molecular mechanisms responsible for development of withdrawal manifestations have been intensely studied, and it has been found that abrupt cessation of BZD administration results in dysregulation of both the GABAergic and glutamatergic transmitter systems. Long term administration of GABAergic drugs leads to compensatory changes in the glutamatergic system because the brain attempts to restore excitatory–inhibitory balance. Mechanisms responsible for compensatory changes have been intensely studied and results most of studies revealed NMDA and AMPA receptors, as well as their function, are dysregulated during the withdrawal period. The predominant role of NMDA receptors in withdrawal response was documented decades ago (Steppuhn and Turski, 1993; Tsuda et al., 1998a; b), and the protective effects of NMDA antagonists against withdrawal phenomena have been repeatedly reported (for rev. Podhorna, 2002). Moreover, the dysregulation of AMPA receptors has been repeatedly reported in the context of benzodiazepine withdrawal in adult animals. The extant literature suggests that benzodiazepine withdrawal is associated with upregulation of AMPA receptor binding (for review, see Allison and Pratt, 2003) and GluA1 receptor subunit expression (Das et al., 2008; Song et al., 2007). A number of studies have also demonstrated that AMPA receptor blockade can mitigate the manifestation of benzodiazepine withdrawal (Steppuhn and Turski, 1993; Souza-Pinto et al., 2007). The pivotal function of the GABAergic system in the context of benzodiazepine withdrawal and its associated symptoms has been previously documented. The reduction in the threshold of seizures induced by GABAergic antagonists such as bicuculline or PTZ has been observed in previous studies, as well as in the present study (Kovacevic et al., 2014; Loscher and Honack, 1992; Dolin et al., 1990). The exposure to and withdrawal from BDZs has been associated with rapid, highly dynamic, and region-specific changes in GABA_A_ receptor subunit expression and binding (for review, see Uusi-Oukari and Korpi, 2010). The focus of research in this field is primarily on adult rodents, with the understanding of developmental particulars in this species being somewhat limited. With the identical protocol as employed in this study, our preceding results have showed a remarkable dysregulation of both glutamatergic and GABAergic receptor expression and binding within the period of increased vulnerability to PTZ (i.e., approximately 1 week following CZP cessation) (Kubova et al., 2018; Kubova et al., 2020), thereby suggesting that alterations in these two predominant neurotransmitter systems play a pivotal role in the adaptation to the potentiation of GABAergic transmission and the development of withdrawal in both immature and adult brain, despite the fact that molecular mechanisms can differ considerably through brain maturation. It is noteworthy that alteration in PTZ-sensitivity was observed in adult animals exposed to CZP during early life, i.e., two months after the cessation of treatment. In comparison with vehicle-treated siblings, RMA epochs induced with an additive dose of PTZ were more prevalent in rats experiencing CZP. As we have shown in earlier studies that used the same design, changes to molecules were identified long after CZP exposure had stopped. Using autoradiography, a decline in benzodiazepine receptor binding was observed in most brain structures, along with a decrease in NMDA binding in multiple cortical areas (Kubova et al., 2018; Kubova et al., 2020). This observation was made 2 months after the termination of CZP administration and suggests a potential modification of PTZ-target structures. As demonstrated by Ben-Ari (1997), both the GABAergic and glutamatergic systems play an irreplaceable role in brain maturation and the formation of brain circuitry. Furthermore, as demonstrated by Melamed et al. (2014), even restricted intervention during a period critical to GABAergic system development can perturb the subsequent development and final structure of brain circuitry. Therefore, changes in seizure susceptibility may reflect not only dysregulation of neurotransmission but also disturbances in final structure of neural circuitry with long-term impact on brain function.

Taken together, the results of our experiments on infant rats exposed to clonazepam demonstrate temporary and permanent alterations in neurotransmission systems and brain function. Animals exposed to CZP during a period of brain growth exhibited mild cognitive impairment, significant alterations in social behavior, and shifts in seizure susceptibility, as well as dynamic changes in GABAergic and glutamatergic receptors, both immediate and permanent (Mikulecka et al., 2014a; Mikulecka et al., 2014b; Kubova et al., 2018; Kubova et al., 2020). Animals were exposed to CZP and CZP withdrawal during highly vulnerable period of development, covering period of growth spurt and increased synaptic plasticity (Dobbing and Smart, 1974; Semple et al., 2013; Lohmann and Kessel, 2014) in order to create synaptic network and to process properly environmental stimuli. The results of the present study demonstrate, for the first time, an increase in seizure susceptibility after CZP cessation in infantile rodents. Interestingly, an increase in susceptibility to PTZ was observed even 2 months after CZP exposure ended. Changes in GABAergic and glutamatergic receptor composition, together with other alterations in neurotransmission previously described due to early-life BZDs exposure (Raol et al., 2005) suggest a more complex and dynamic dysregulation in these two systems, which play a key role in neuronal survival, synaptogenesis, and the maturation of synaptic networks and connectivity with impact on the integrity of the neural system (Wadhwa et al., 2025). Data from preclinical studies further demonstrated that BZDs administration during early postnatal development interferes with cell survival and neurogenesis (Bittigau et al., 2002; Stefovska et al., 2008; Olney et al., 2002; Frederiksson et al., 2004; Chen et al., 2009). Taken together, all these effects result in immediate and, in some instances, long-lasting neural alterations (see Andersen and Navala, 2004, for a review).

## 6 Conclusion

Our results demonstrate development of withdrawal increase of seizure susceptibility as well as permanent shift in sensitivity to PTZ in rat exposed to CZP during a highly vulnerable period of brain maturation, covering the growth spurt and increased synaptic plasticity. We hypothesise that even transient disruption to the delicate balance between the two neurotransmitter systems critically involved in the maturation of neural networks as documented before (Kubova et al., 2018; Kubova et al., 2020) could be responsible for the functional consequences observed in our studies, as well as in those of others. Our results also document the development of increased seizure susceptibility after even short-lasting treatment with CZP. Together with our observation of a rebound response after a single dose of CZP (Mikulecka et al., 2011), this suggests that immature individuals may be more prone to increased excitability after abrupt benzodiazepine (BZD) cessation. These results are consistent with clinical observations showing that children are more susceptible to developing withdrawal syndrome after benzodiazepine termination (for rev, Authier et al., 2009). In addition to the heightened risk of other adverse effects associated with benzodiazepine administration in children like respiratory depression, hypotension and neurological abnormalities (for rev Ng et al., 2002), the increased propensity for withdrawal syndrome should be considered when selecting a therapeutic regimen in pediatric patients.

## Data Availability

The raw data supporting the conclusions of this article will be made available by the authors, without undue reservation.
