# Supplementary material for: Early life exposure to clonazepam has both short- and long-term effects on seizures induced with pentylenetetrazol (PTZ)
Source: Front Pharmacol. 2026 Jan 13;16:1725780. doi: 10.3389/fphar.2025.1725780 (PMC12835315; doi:10.3389/fphar.2025.1725780)
Supplement: Supplementary file 1 [file DataSheet1.pdf]

Supplementary materials

Supplementary table A. *Relative body weight gain*. Body weight at P6 was normalized to 100% and differences in relative body weight between two consecutive days was expressed in %. The relative body weight gains were analyzed with Mixed effects analysis followed by FDR-corrected multiple comparison tests (q< 0.05 taken as discovery). C – controls (upper row); CZP – treated (lower row).

total number of control animals = 86, total number of animals in the CZP group = 75.

Post-hoc Two-stage linear step-up procedure of Benjamini, Krieger and Yekutieli<br>Relative weight gain (%)<br>(mean ± SD)<br>C<br>*vs.*<br>CZP<br>*(q = discovery)*		
Factor	Mixed effects analysis<br>F (DFn, DFd)	P value
Days	F (5.089; 170.5) = 47.8	<0.0001
Treatment	F (1.40) = 6.376	0.0156
Days x treatment	F (5.089; 170.5) = 7.836	<0.0001

P6/7	P7/8	P8/9	P9/10	P10/11	P11/12	P12/13	P13/14	P14/15
16.6<br>±2.1<br>*vs.*<br>16.3<br>±2.2	15.3<br>±2.3<br>*vs.*<br>9.5<br>±3.2	14.7<br>±2.5<br>*vs.*<br>12.3<br>±3.6	11.3<br>±1.9<br>*vs.*<br>9.9<br>±2.0	9.0<br>±4.5<br>*vs.*<br>9.4<br>±2.7	8.8<br>±4.5<br>*vs.*<br>8.4<br>±2.1	9.2<br>±1.6<br>*vs.*<br>9.9<br>±2.3	7.2<br>±1.6<br>*vs.*<br>8.6<br>±2.7	7.5<br>±1.4<br>*vs.*<br>7.7<br>±1.9
(0.5976)	*(<0.0001)*	*(0.0234)*	*(0.1205)*	*(0.5976)*	*(0.5976)*	*(0.5976)*	*(0.1625)*	*(0.5976)*

(B) Immediate increase of seizure susceptibility after CZP cessation - mS and GTCS seizures induced with single, threshold dose of PTZ

Supplementary table B1*. Severity of motor seizures expressed as score*. PTZ was administered subcutaneously in a single threshold dose of 50 mg/kg in P13, P15 and P18 animals and in a dose of 60 mg/kg in P21 and P25 animals. Analysis did not reveal any significant age-dependent differences in seizure severity among vehicle treated animals. Early CZP exposure significantly increased the severity of seizures induced after treatment cessation until P18 (i.e. seven days after the last CZP injection). Behavioral seizure scores were analyzed using Kruskal-Wallis test corrected for multiple comparisons by controlling the False Discovery Rate of Benjamini, Krieger, and Yekutieli (discovery $q < 0.05$). Data presented as median and 25% and 75% percentile. C – controls (upper row); CZP – treated (lower row), n_C – number of animals in the control group, n_{CZP} – number of animals in the CZP group.

Kruskal-Walli's test (K-W statistics)	P value	Score<br>(median, 25% and 75% percentile)<br>*Post-hoc* Two-stage linear step-up procedure of Benjamini, Krieger and Yekutieli<br>C<br>*vs.*<br>CZP<br>*(q = discovery)*				
		P13<br>$n_C = 12$<br>$n_{CZP} = 8$	P15<br>$n_C = 14$<br>$n_{CZP} = 8$	P18<br>$n_C = 16$<br>$n_{CZP} = 18$	P21<br>$n_C = 8$<br>$n_{CZP} = 8$	P25<br>$n_C = 8$<br>$n_{CZP} = 8$
39.23	<0.05	1.000<br>(1.000, 2.000)<br>*vs.*<br>5.000<br>(4.250, 5.000)	2.000<br>(1.000, 2.250)<br>*vs.*<br>4.000<br>(4.000; 4.000)	1.000<br>(1.000; 2.000)<br>*vs.*<br>3.000<br>(1.250; 4.000)	1.000<br>(1.000; 5.000)<br>*vs.*<br>1.000<br>(1.000; 2.500)	1.000<br>(1.000; 1000)<br>*vs.*<br>1.000<br>(1.000; 1.000)
		q = 0.0006	*q = 0.0422*	*q = 0.0422*	*q = 0.3234*	*q = 0.8400*

Supplementary table B2. *Incidence of GTCS* (%). Seizure incidence was evaluated using Fisher's exact test (level of significance $p < 0.05$). The incidence of GTCS was very low and did not differ between age groups (Chi-squared test: 1.723, df = 3; $p > 0.05$). Early CZP treatment significantly increased the incidence of GTCS (Chi-squared test: 30.35, df = 12; $p = 0.0025$) until P18, as revealed by Fisher's exact test. The incidence of GTCS decreased over time and, beginning on P21, it was not different from the vehicle-treated group. Data presented in %. C – controls (upper row); CZP – treated (lower row), n_C – number of animals in the control group, n_{CZP} – number of animals in the CZP group.

Incidence GTCS (%)<br>Fisher's exact test<br>C<br>*vs.*<br>CZP<br>*(P value)*				
P13	P15	P18	P21	P25
16.7 vs. 100%	21.4 vs. 87.5%	8.7 vs. 43.8%	25 vs. 12.5%	0 vs. 0%
(0.0007)	*(0.0062)*	*(0.0019)*	*(>0.9999)*	*(>0.9999)*

(C) Short- and long-term changes in susceptibility to Rhythmic Metrazol Activity (RMA) and myoclonic seizures induced with additive dose of PTZ

Supplementary table C1. *Number of RMA epochs* in the 1st and 2nd evaluated interval after the PTZ administration. PTZ was administered intraperitoneally at P18, P25 or P90 in three consecutive doses of 20 mg/kg with an interval of 20 minutes between the injections or until the onset of myoclonic seizures in animals with implanted silver recording electrodes. The number of RMA epochs was evaluated between 10 and 15 min after the first and second PTZ injections. Data was analyzed using Two-way repeated measures ANOVA with one between-group factor (treatment) and one within-subject factor (repeated session) followed by FDR-corrected multiple comparison tests (q< 0.05 taken as discovery). In controls, number of RMA epochs was dependent on the age of the animals on the day of recording and the total dose of PTZ (i.e. the number of the recording session). In P90 animals, the number of RMA epochs increased five times between the 1st and 2nd evaluated intervals. In the first evaluated interval, RMA episodes were significantly more frequent in both groups of juveniles compared to P90 rats (P18 *vs.* P90, q=0.00225; P25 *vs.* P90, q=0.0303). As demonstrated in table below, early life CZP exposure tended to increase number of RMA epochs compared to controls in the 1st interval in P18 and P25 rats, but significant difference was observed only in P90 animals. No differences in the number of RMA epochs between controls and CZP exposed animals were observed in the 2nd interval.

Data presented in mean ± SD. C – controls (upper row); CZP – treated (lower row), n_C – number of animals in the control group, n_{CZP} – number of animals in the CZP group.

Factor	RM One-way ANOVA (treatment)<br>F (DFn, DFd)	P value	Interval	*Post-hoc* Two-stage linear step-up procedure of Benjamini, Krieger and Yekutieli<br>Number of RMA; mean ±SD<br>*(q = discovery)*			
				1st interval C *vs*. CZP	2nd interval C *vs*. CZP	1st *vs* 2nd interval C	1st *vs* 2nd interval CZP
Interval	F (1,4) = 13.05	P=0.0008	P18<br>n_C= 10<br>n_{CZP}= 9	16.7±10.4<br>vs<br>8.8±5.0<br>*(0.0769)*	6.7±5.6<br>vs<br>7.4±7.5<br>*(0.4718)*	16.7±10.4<br>vs<br>6.7±5.6<br>*(0.0055)*	8.8±5.0<br>vs<br>7.4±7.5<br>*(0.6652)*
Treatment<br><br>Interval x treatment	F (5, 43) = 3.177<br><br>F (5, 43) = 10.43	P=0.0158<br><br>P<0.0001	P25<br>n_C = 8<br>n_{CZP}= 8	14.5±5.7<br>vs<br>6.2±2.5<br>*(0.0657)*	20.6±10.0<br>vs<br>13.3±7.7<br>*(0.0609)*	14.5±5.7<br>vs<br>20.6±10.0<br>*(0.0457)*	6.3±2.5<br>vs<br>13.3±7.7<br>*(0.0170)*
			P90<br>n_C= 10<br>n_{CZP}= 8	4.5±2.5<br>vs<br>13.7±7.2<br>*(0.0313)*	23.3±10.9<br>vs<br>19.5±8.3<br>*(0.1970)*	4.5±2.6<br>vs<br>23.3±10.9<br>*(<0.0001)*	13.7±7.2<br>vs<br>19.5±8.3<br>*(0.0603)*

Supplementary table C2. *Latency to the 1st RMA after the 1st PTZ administration*. Data was analyzed using One-way ANOVA followed by FDR-corrected multiple comparison tests. In controls, latencies to the 1st RMA epoch were significantly shorter in both groups of juvenile rats compared to P90 animals (P18 *vs.* P90 q<0.0001; P25 *vs.* P90 q<0.0001). Early life exposure to CZP resulted in significant

shortening of latency to the 1st RMA epoch in P90 rats but did not affect this parameter in juvenile animals.

Data presented in mean ± SD. C – controls (upper row); CZP – treated (lower row), n_C – number of animals in the control group, n_{CZP} – number of animals in the CZP group.

Factor	One-Way ANOVA F (DFn, DFd)	P- value	Age at PTZ administration	*Post-hoc* Two-stage linear step-up procedure of Benjamini, Krieger and Yekutieli *(q = discovery)*
				The 1st RMA latency (s), mean±SD C vs. CZP (*q*)
Treatment	F (5, 43) = 10.16	<0.0001	P18 n_C= 10; n_{CZP}= 9	77 ± 111 *vs.* 131 ± 66 (*0.3351*)
			P25 n_C = 8; n_{CZP}= 8	32 ± 35 *vs.* 68 ± 48 (*0.3907*)
			P90 n_C = 10; n_{CZP}=8	400 ± 242 *vs.* 200 ± 92 (*0.0037*)

Supplementary table C3. *Total duration of RMA epochs* in the 1st and 2nd evaluated interval after the PTZ administration. Total duration of RMA was compared with Two-way ANOVA RM followed by FDR-corrected multiple comparison tests. Table below shows that early life exposure to CZP resulted to significant increase of total duration of RMA activity compared to controls in the 1st evaluated interval. In addition, statistical analysis revealed significant increase of total duration of RMA activity in P90 rats between the 1st and 2nd interval in both control and CZP exposed animals.

Data presented in mean ± SD. C – controls (upper row); CZP – treated (lower row), n_C – number of animals in the control group, n_{CZP} – number of animals in the CZP group.

Factor	RM One-way ANOVA (treatment) F (DFn, DFd)	P value	Interval	*Post-hoc* Two-stage linear step-up procedure of Benjamini, Krieger and Yekutieli Total duration of RMA (s); mean ±SD *(q = discovery)*			
				1st interval C *vs.* CZP	2nd interval C *vs.* CZP	1st *vs* 2nd interval C	1st *vs* 2nd interval CZP
Interval	F (1, 43) = 13.93	P=0.0006	P18 n_C= 10 n_{CZP}= 9	15.2±11.2 vs 11.8±8.6 *(0.6782)*	5.6±4.3 vs 10.1±15.6 *(0. 3953)*	15.2±11.2 vs 5.6±4.2 *(0.4769)*	11.8±8.6 vs 10.1±15.6 *(0.9151)*
Treatment	F (5, 43) = 10.97	P<0.0001	P25 n_C = 8 n_{CZP}= 8	10.0±3.8 vs 4.9±2.7 *(0.6782)*	17.9±11.5 vs 10.9±7.7 *(0.3953)*	10.1±3.8 vs 17.9±11.5 *(0.5015)*	4.9±2.7 vs 10.9±7.7 *(0.6139)*
Interval x treatment	F (5, 43) = 7.591	P<0.0001	P90 n_C= 10 n_{CZP}= 8	11.6±9.0 vs 59.1±43.6 *(0.0030)*	80.2±47.1 vs 85.2±58.9 *(0.3953)*	11.6±9.0 vs 80.2±47.1 *(<0.0001)*	56.1±43.6 vs 85.2±58.9 *(0.0115)*

Supplementary table D. *Latency to the 1st myoclonic seizure*. Latencies to the onset of the 1st mS were analyzed using One-way ANOVA followed by FDR-corrected multiple comparison tests (q< 0.05 taken as discovery).

Data presented in mean ± SD. C – controls (upper row); CZP – treated (lower row), n_C – number of animals in the control group, n_{CZP} – number of animals in the CZP group.

Factor	One way ANOVA F (DFn, DFd)	P value	Age	*Post-hoc* Two-stage linear step-up procedure of Benjamini, Krieger and Yekutieli *(q = discovery)* mS latency (s); mean ±SD C *vs*. CZP
Treatment	F (5, 38) = 17.71	<0.0001	P18 n_C= 10; n_{CZP}= 9	1491 ± 619 *vs*. 665 ± 310 *(0.0031)*
			P25 n_C = 8; n_{CZP}= 8	1391 ± 695 *vs*. 1566 ± 852 *(0.1765)*
			P90 n_C = 10; n_{CZP}=8	2561 ± 92 *vs*. 2957 ± 434 *(0.0656)*
